# Evaluating the efficacy of vestibular rehabilitation therapy on quality of life in persistent postural-perceptual dizziness: the role of anxiety and depression in treatment outcomes

**DOI:** 10.3389/fneur.2025.1524324

**Published:** 2025-02-04

**Authors:** Khalid A. Alahmari, Sarah Alshehri

**Affiliations:** ^1^Program of Physical Therapy, Medical Rehabilitation Sciences, College of Applied Medical Sciences, King Khalid University, Abha, Saudi Arabia; ^2^Otology and Neurotology, Department of Surgery, College of Medicine, King Khalid University, Abha, Saudi Arabia

**Keywords:** dizziness, vestibular rehabilitation therapy, quality of life, anxiety, depression

## Abstract

**Background:**

Persistent Postural-Perceptual Dizziness (PPPD) is a chronic disorder that significantly affects the quality of life (QoL) and daily living. Vestibular rehabilitation therapy (VRT) has emerged as a promising treatment option, yet its effectiveness, particularly in relation to symptom duration, anxiety, and depression, remains underexplored.

**Methods:**

This cross-sectional study assessed the effect of VRT on the QoL in 188 PPPD patients, as well as the correlation between the duration of symptoms and dizziness severity and the role of anxiety and depression in the treatment response. QoL was assessed using the EuroQol-5 Dimension (EQ-5D), and dizziness-related handicap using the Dizziness Handicap Inventory (DHI) and anxiety and depression using the Hospital Anxiety and Depression Scale (HADS).

**Results:**

Ninety three patients were included in the analysis. QoL was significantly improved post-VRT with a greater mean EQ-5D score (EQ-5D post-VRT 0.72; EQ-5D pre-VRT 0.61, *p* = 0.032). Age was also found to correlate with increased dizziness severity (*p* < 0.001), although this was not as strong as the correlation of symptom duration (longer duration correlating with increased dizziness severity; *p* < 0.01), anxiety (*r* = −0.45, *p* = 0.002) and depression (*r* = −0.51, *p* < 0.001) (both significantly associated with poorer outcomes especially with reference to VRT) emerging as independent correlates of reduced effectiveness of treatment.

**Conclusions:**

This study revealed the benefit of VRT in patients with PPPD on QoL and indicates the importance of identifying and treating psychological factors to improve the success of treatment for PPPD.

## 1 Introduction

Persistent Postural-Perceptual Dizziness (PPPD) is a chronic vestibular syndrome associated with continuous dizziness or imbalance exacerbated by visual stimuli, movement, and body position changes (1). These symptoms can impair activities of daily living and markedly decrease quality of life (QoL) (2). While the precise pathophysiology remains poorly understood, the prevailing hypothesis is that PPPD develops due to maladaptive interactions between the central vestibular system, the visual system, and the proprioceptive system, exacerbated by psychological factors including anxiety and hypervigilance (3). This interaction commonly results in a cycle of persistent symptoms, functional impairments, and augmented psychological distress.

Vestibular rehabilitation therapy (VRT) is a new treatment used to manage PPPD (4). The goal of VRT is to enhance vestibular compensation and neuroplasticity by focusing on gaze stabilization, postural control, and sensory integration (5). Although studies showed its effectiveness in the reduction of dizziness and enhancement of functional performance in people with general vestibular disorders, evidence assessing its effect on PPPD is limited, and even less is known about the quality of life (QoL) effect of RE in these patients (6). Because PPPD is such a chronic and multifactorial condition, exploring how VRT improves dizziness and well-being in this population is of the utmost importance (7).

They may play a major role in the outcomes of the VRT process: symptom duration and psychological comorbidities (anxiety and depression) (8). Prolonged patients often develop maladaptive coping mechanisms, which, in turn, lead to functional limitations and reduce rehabilitation efficacy (9). Anxiety and depression add to the treatment burden, with the former increasing sensitivity to vestibular stimuli and the latter reducing motivation for therapy. Despite these limitations, the interaction between the chronicity of symptoms, mental health variables, and the outcomes of VRT remains under-explored (10).

Current studies on VRT for vestibular disorders do not consider the specific features of PPPD, and many studies focus on immediate improvement without clarifying the effects of symptom duration or psychological characteristics (9). Hence, this study will address these gaps by examining the effects of VRT on QoL and dizziness-related handicaps in patients with PPPD, paying specific attention to the role of symptom duration, anxiety, and depression (10). The objectives are to (1) determine the association between VRT and the QoL of adults diagnosed with PPPD, (2) analyze the association of symptom duration in PPPD with dizziness-related handicap scores in individuals receiving VRT for their condition, and (3) analyze the impact of anxiety and depression on the effectiveness of VRT in reducing symptoms as well as disability in adults with PPPD.

## 2 Materials and methods

### 2.1 Study design, ethics, and settings

The Vestibular and Balance Disorders Clinic, KKU Medical City (KKUMC), Saudi Arabia, is a specialized unit embedded into a primary care hospital, in line with a cross-sectional study design between March 22, 2023, and February 12, 2024. As this center accommodates patients referred by general practitioners and other healthcare providers, the cohort of participants surveyed included a wide range of PPPD severities (early stage, chronic) in accordance with the above definitions. This study was approved by an ethical review board and conducted in accordance with the Declaration of Helsinki principles. King Khalid University Institutional Review Board (IRB) provided ethical approval (REC# 345-2023). All participants provided written informed consent prior to enrollment, which confirmed their understanding of the study objectives and procedures and their right to withdraw at any time without repercussions.

### 2.2 Participants

Participants for this study were adults aged 18–65 years who were clinically diagnosed with PPPD based on the Bárány Society criteria (11). The diagnosis was confirmed by a neuro-otologist following a comprehensive assessment that included a detailed medical history, physical examination, and relevant vestibular function tests such as videonystagmography (VNG) and vestibular evoked myogenic potentials (VEMPs) to rule out other vestibular or neurological disorders (11, 12). The inclusion criteria were as follows: (1) a confirmed diagnosis of PPPD, characterized by patients often experiencing non-spinning vertigo alongside dizziness and unsteadiness, as described by the Bárány Society criteria; (2) willingness to participate in a VRT program; (3) the ability to comprehend and complete self-reported questionnaires, including the DHI, Hospital Anxiety and Depression Scale (HADS), and EQ-5D; and (4) stable medical conditions with no recent changes in medication related to dizziness.

Exclusion criteria were as follows: (1) the presence of other active vestibular disorders, such as Meniere's disease, vestibular migraine, or benign paroxysmal positional vertigo (BPPV); (2) neurological or psychiatric disorders that could confound the results, such as multiple sclerosis or severe depression; (3) ongoing use of vestibular-suppressant medications within the past 30 days; (4) prior participation in VRT or any form of physical therapy for dizziness within the last 6 months; (5) significant visual or cognitive impairments that would hinder participation in rehabilitation exercises or completing questionnaires; and (6) pregnancy, as hormonal changes may influence dizziness symptoms.

Symptom duration was a key variable, and subgroup analyses were planned to evaluate differences in outcomes between patients with symptoms lasting < 1 year and those lasting more than 1 year. To ensure consistency in diagnosis and the administration of VRT, clinical assessments were conducted by a team of experienced healthcare professionals, including neuro-otologists and physical therapists specialized in vestibular disorders. Inter-rater reliability was assessed to ensure uniformity in the evaluation of participants and the administration of VRT exercises. Calibration sessions standardized the use of assessment tools, including the DHI, HADS, and EQ-5D, ensuring uniform scoring and interpretation among all assessors. These sessions involved practice evaluations, scoring comparisons, and resolution of discrepancies through consensus. This process was integral to maintaining high inter-rater reliability, as reflected by the intraclass correlation coefficient (ICC) of 0.85. The DHI (13), HADS (14), and EQ-5D (15) instruments used in this study were validated Arabic versions, which are widely recognized and previously established for use in Arabic-speaking populations.

### 2.3 Vestibular rehabilitation therapy

The VRT protocol used in this study was tailored to address the specific symptoms and functional impairments of patients with PPPD (7). The VRT program consisted of a combination of exercises targeting gaze stabilization, habituation, and postural stability (16). Gaze stabilization exercises were designed to improve visual fixation during head movements, typically involving repeated eye and head movements while focusing on a stationary target (16). Habituation exercises were used to reduce dizziness and sensitivity to movement, involving repeated exposure to motions or environments that triggered symptoms, allowing the brain to adapt gradually (16). Postural stability training included balance exercises, such as standing on different surfaces or performing tasks that challenged the vestibular system, aimed at improving overall balance and reducing fall risk (17).

The therapy sessions were conducted both in the clinic and at home. Participants attended supervised clinic sessions twice a week, with each session lasting ~45 min. In addition, participants were provided with a home-based exercise program to be performed daily, which took ~20–30 min to complete. Compliance with the home-based program was monitored through patient logs, and adjustments to the exercise regimen were made as needed based on individual progress. The entire VRT intervention spanned a period of 8 weeks, with assessments conducted at baseline and at the end of the therapy period to measure treatment outcomes.

Uptake of previously cited benefits of VRT, a commonly employed treatment for PPPD, have recently been compared both pre- and post-intervention to ascertain whether and how much VRT improves QoL and decreases dizziness-related handicaps. To put the changes into context, a non-VRT observational group of patients before the VRT program was included as a comparator. This group included patients with PPPD who had similar baseline characteristics but did not receive VRT during the same period. Adding this observational cohort to facilitate the evaluation of treatment effects without therapy highlighted the relative effectiveness of VRT. Although it lacks prospective matching, the observational group provides insight into the natural history of PPPD and the potential role of VRT as a potential treatment in attenuating symptoms. This design allows for a comprehensive evaluation of treatment outcomes while also aligning with the constraints of practical randomization.

### 2.4 Variables

#### 2.4.1 Quality of life

The primary outcome variable in this study was QoL, which was measured using the EQ-5D questionnaire, a standardized instrument widely used for assessing health-related QoL (18). The EQ-5D generates a single index score ranging from 0 (worst imaginable health state) to 1 (perfect health), reflecting the participant's self-reported overall well-being across five domains: mobility, self-care, usual activities, pain/discomfort, and anxiety/depression (19). This score was recorded both pre-and post-VRT to evaluate the impact of VRT on participants' QoL (19).

#### 2.4.2 Dizziness handicap inventory

The secondary outcome variable was a dizziness-related handicap, measured by the DHI (20). The DHI is a validated, self-administered questionnaire used to quantify the physical, emotional, and functional impacts of dizziness (21). It consists of 25 items, each scored on a scale from 0 to 4, with a total score ranging from 0 (no handicap) to 100 (severe handicap) (21). Higher scores indicate a greater dizziness-related handicap (21). This measure was used to assess the severity of dizziness and how it affected patients' daily activities before and after VRT.

#### 2.4.3 Hospital anxiety and depression scale

Anxiety and depression were also key variables, assessed using the HADS, which consists of two subscales: one for anxiety and one for depression, with seven items each. Each item is scored on a 4-point scale (0–3), with total subscale scores ranging from 0 to 21 (22). A score of 0–7 indicates normal levels of anxiety or depression, 8–10 indicates borderline abnormal, and 11–21 indicates clinically significant levels. HADS was used to evaluate the psychological status of participants both before and after VRT, with higher scores reflecting greater levels of anxiety and depression (22).

#### 2.4.4 Additional variables

Additional variables included symptom duration, defined as the length of time participants had experienced symptoms of PPPD prior to starting VRT. Symptom duration was categorized as < 1 year or more than 1 year, based on patient self-report during initial assessment. The relationship between symptom duration and the severity of dizziness-related handicap (DHI score) was analyzed to determine whether the chronicity of symptoms influenced VRT outcomes.

### 2.5 Follow-up and outcome measurement

Outcome measurements were conducted at two time points: baseline (pre-VRT) and immediately after the completion of the vestibular rehabilitation therapy (post-VRT), which spanned a total of 8 weeks. At baseline, participants completed the DHI, HADS, and EQ-5D to assess dizziness-related handicap, psychological status, and QoL, respectively. These same measures were repeated at the end of the 8-week VRT program to evaluate the effect of therapy. In addition to pre- and post-intervention assessments, a follow-up evaluation was conducted at 12 weeks post-therapy to assess the sustainability of treatment effects on QoL and dizziness-related handicap.

### 2.6 Covariates

Other covariates included age, gender, and comorbid conditions (e.g., hypertension, diabetes), which were collected through patient medical records and structured interviews. These variables were included as potential confounders in the analysis, given their possible influence on treatment outcomes and overall health status. All data were collected *via* standardized questionnaires, patient interviews, and clinical assessments conducted by trained healthcare professionals.

### 2.7 Sample size estimation

The sample size was determined using G^*^Power 3.1 statistical software according to a moderate effect size of 0.5, a power of 0.80, and a significance level of 0.05. Based on our sample size calculation, we estimated that a sample of 188 participants would be adequate to detect clinically meaningful differences in QoL measures (DHI, EQ-5D, and HADS scores). The sample size meditated for planned subgroup analyses based on symptom-onset duration (1 year) and separates between VRT and retrospective non-VRT observational groups to warrant adequate information to determine statistically important variants in QoL and dizziness out-planned (31, 32). This also accounted for possible subgroup analyses (stratification depending on symptom duration) and multivariate adjustments for confounding factors, including age, anxiety, and depression. Considering an estimated 10% rate of incomplete or invalid responses, the target sample size was calculated to provide sufficient power to detect statistically significant associations between vestibular rehabilitation and the respective outcomes of interest.

### 2.8 Data analysis

Data was analyzed using SPSS software version 24. Descriptive statistics, including means and standard deviations, were used to summarize continuous variables, while frequencies and percentages summarized categorical variables. Missing or incomplete data were addressed using listwise deletion, excluding participants with missing data on key outcome variables from the analysis. To assess the relationship between VRT and QoL, independent *t-*tests compared mean EQ-5D scores between participants with varying durations of PPPD symptoms. One-way ANOVA was employed to evaluate the association between symptom duration and dizziness severity, specifically comparing DHI scores across symptom duration groups. Subgroup analyses further explored differences in DHI scores based on symptom duration. Pearson correlation analysis was conducted to investigate the relationships between mental health outcomes (HADS scores) and DHI scores. Multiple linear regression models were used to determine the independent effects of anxiety and depression on outcomes while adjusting for confounders such as age, gender, and comorbidities. The inclusion of these covariates was informed by prior literature and significant univariate associations (*p* < 0.10) identified during preliminary analyses. Sensitivity analyses were also performed to evaluate the consistency and robustness of the regression models when incorporating different sets of covariates.

## 3 Results

The demographic and clinical characteristics of the VRT and retrospective non-VRT observational groups are presented in Table 1. The retrospective non-VRT observational group served as an observational cohort for comparison. Although they did not undergo VRT, baseline characteristics, and clinical outcomes were assessed to evaluate the relative effectiveness of VRT. The groups were comparable in terms of age and gender distribution, with no significant differences observed (p > 0.05). However, a higher proportion of participants in the VRT group had a symptom duration of < 1 year compared to the retrospective non-VRT observational group (54.26% vs. 48.94%, *p* = 0.023). Clinically, the VRT group showed significantly lower mean DHI, HADS Anxiety, and HADS Depression scores compared to the retrospective non-VRT observational group (*p* < 0.01 for all), indicating less severe dizziness-related handicap and better mental health outcomes. Additionally, the VRT group had a higher mean EQ-5D score, reflecting better QoL (*p* = 0.032).

**Table 1:** Demographic and clinical characteristics.

**Characteristic**	**VRT Group (*n =* 94)**	**Retrospective non-VRT observational group (*n =* 94)**	***p-*value**
Age (years)	45.32	46.12	0.235
Male (%)	60.64	58.51	0.412
Female (%)	39.36	41.49	0.412
Symptom Duration < 1 year (%)	54.26	48.94	0.023
Symptom Duration > 1 year (%)	45.74	51.06	0.023
Mean DHI score	38.42	45.61	0.001
Mean HADS anxiety score	10.25	13.58	0.007
Mean HADS depression score	8.73	10.21	0.009
Mean EQ-5D score	0.72	0.61	0.032

DHI, Dizziness handicap inventory; HADS, Hospital anxiety and depression scale; EQ-5D, EuroQol-5 dimension; VRT, Vestibular rehabilitation therapy.

**Figure 1:**
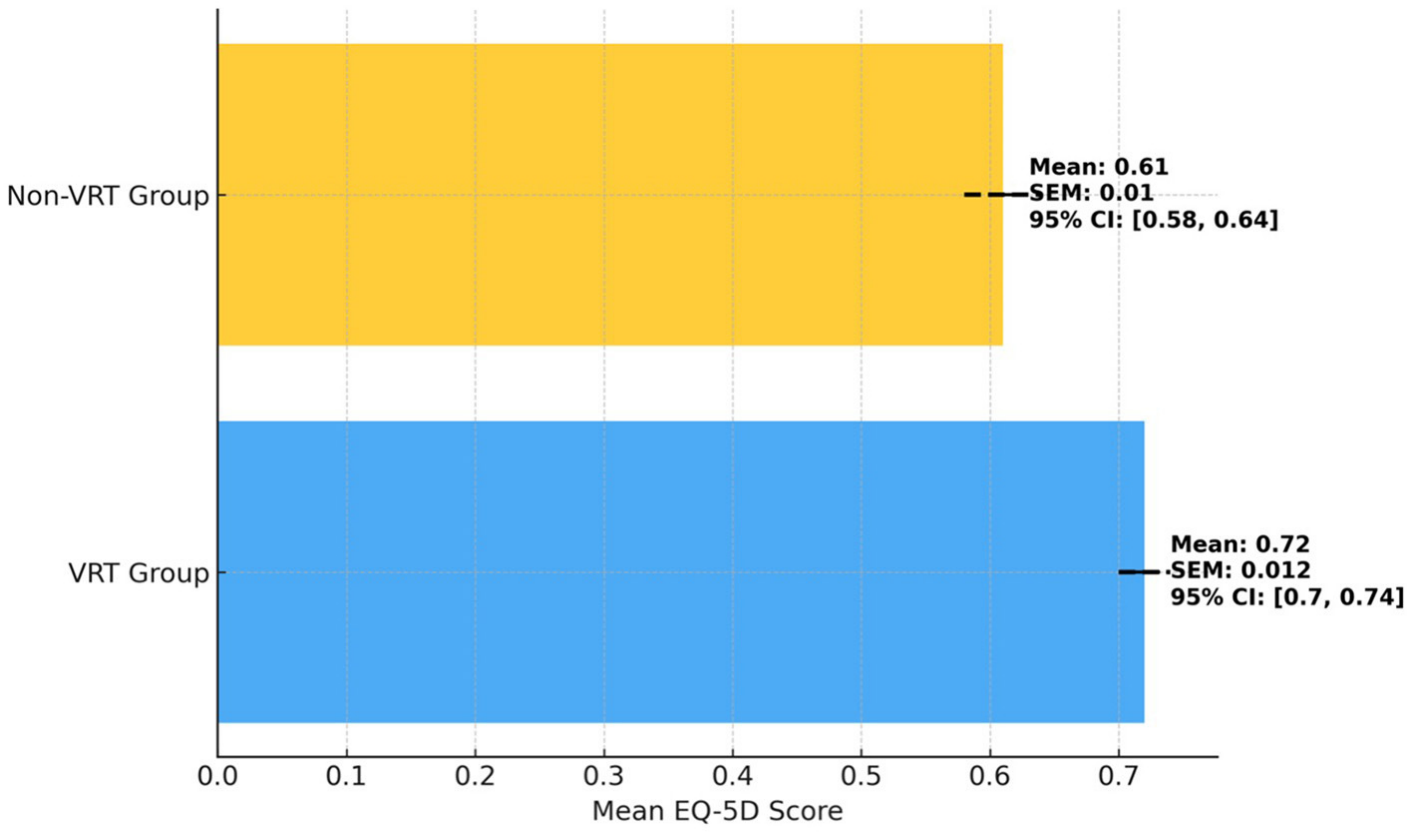
Comparison of EQ-5D scores between VRT and retrospective non-VRT observational groups.

The comparison of EQ-5D scores between the VRT and Retrospective non-VRT observational groups showed a statistically significant difference in favor of the VRT group (Table 2; Figure 1). Patients who underwent vestibular rehabilitation therapy had a higher mean EQ-5D score of 0.72 compared to 0.61 in the Retrospective non-VRT observational group (*p* = 0.032). This indicates that the VRT group experienced a better QoL, as reflected by higher EQ-5D scores. The 95% confidence intervals for the VRT group were between 0.70 and 0.74, while for the Retrospective non-VRT observational group, they ranged from 0.58 to 0.64. This further supports the effectiveness of VRT in improving QoL in patients with PPPD.

**Table 2:** Comparison of EQ-5D scores between VRT and retrospective non-VRT observational groups.

**Group**	**Mean EQ-5D score**	**Standard deviation**	**Standard error of mean (SEM)**	**95% CI (lower)**	**95% CI (upper)**	***t*-value**	**Degrees of freedom (d*f*)**	***p-*value**
VRT Group (*n =* 94)	0.72	0.12	0.012	0.7	0.74	2.17	186	0.032
Retrospective non-VRT observational group (*n =* 94)	0.61	0.1	0.01	0.58	0.64	2.17	186	0.032

EQ-5D, EuroQol-5 dimension; VRT, Vestibular rehabilitation therapy.

**Figure 2:**
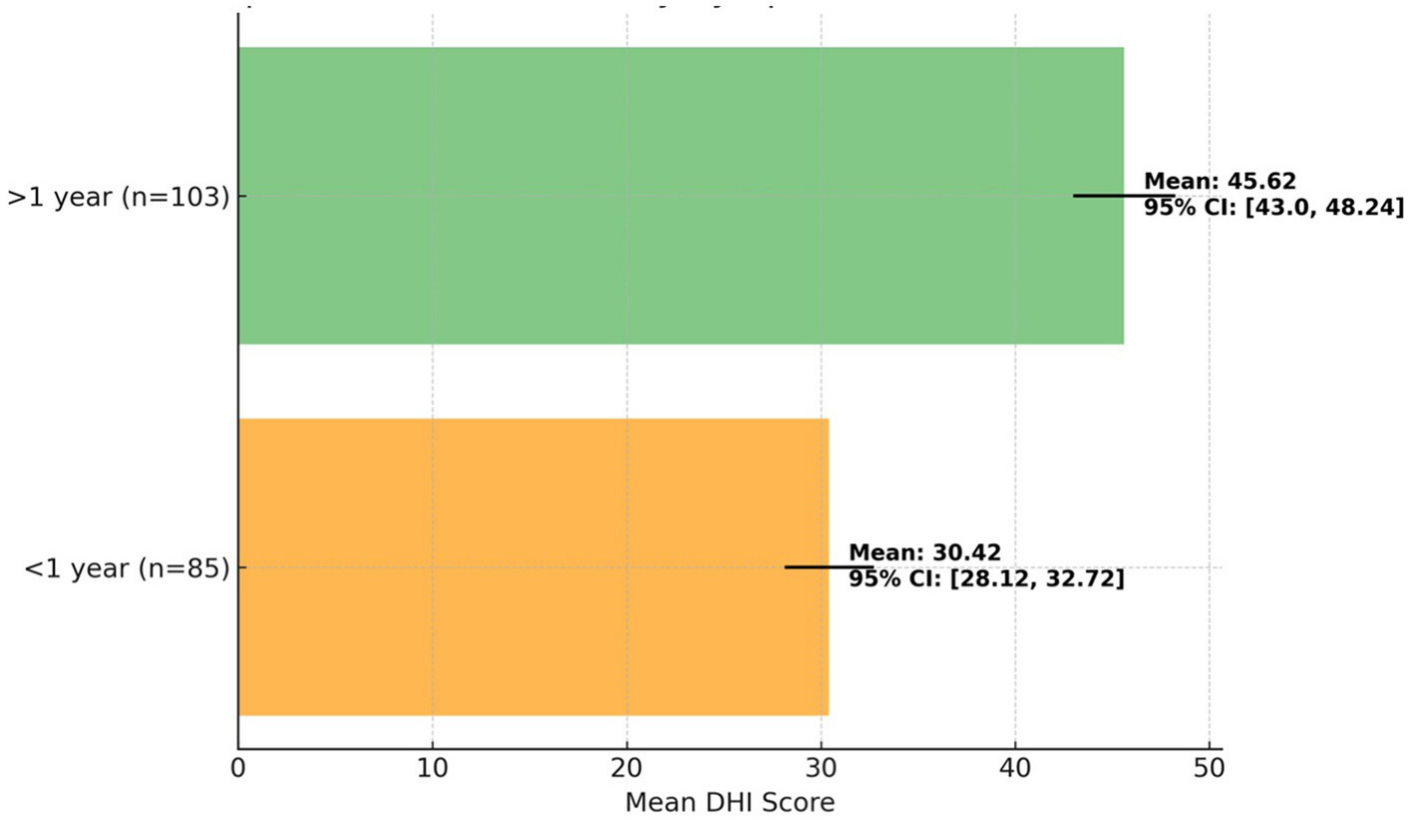
Comparison of DHI scores by symptom duration in VRT patients.

Patients with PPPD symptoms for more than 1 year had significantly higher DHI scores compared to those with symptoms lasting < 1 year (Table 3; Figure 2). Specifically, the >1 year group had a mean DHI score of 45.62, while the < 1 year group had a mean score of 30.42 (*p* < 0.01). This indicates that a longer symptom duration is associated with a greater dizziness-related handicap, suggesting that patients with more chronic symptoms experience more severe impairments in daily functioning. The ANOVA results showed a significant effect of symptom duration on DHI scores, with an *F-*value of 15.67.

**Table 3:** Comparison of DHI scores by symptom duration in VRT patients.

**Symptom duration group**	**Mean DHI score**	**SD**	**95% CI (lower)**	**95% CI (upper)**	***F-*value**	***p-*value**
< 1 year (*n =* 85)	30.42	7.54	28.12	32.72	15.67	0.001
>1 year (*n =* 103)	45.62	9.31	43.0	48.24	15.67	0.002

DHI, Dizziness handicap inventory; VRT, Vestibular rehabilitation therapy.

The subgroup analyses revealed significant differences in DHI and EQ-5D scores based on symptom duration, anxiety, and depression levels (Table 4). Patients with symptom durations of < 1 year had lower DHI scores (mean = 30.42, SD = 7.54) and higher EQ-5D scores (mean = 0.81, SD = 0.12) compared to those with symptoms persisting for more than 1 year (mean DHI = 45.62, SD = 9.31; mean EQ-5D = 0.61, SD = 0.10). Similarly, lower levels of anxiety and depression were associated with better outcomes, as patients with low HADS Anxiety (0–7) and HADS Depression (0–7) scores demonstrated lower DHI scores (mean = 28.45, SD = 6.75; mean = 29.34, SD = 6.85) and higher EQ-5D scores (mean = 0.85, SD = 0.11; mean = 0.84, SD = 0.10) than those with high anxiety (mean DHI = 48.23, SD = 10.34; mean EQ-5D = 0.62, SD = 0.15) or high depression (mean DHI = 49.78, SD = 9.45; mean EQ-5D = 0.60, SD = 0.14). Regression analyses confirmed that higher anxiety and depression levels and longer symptom duration were significantly associated with worse DHI and EQ-5D outcomes, with regression coefficients ranging from −0.20 to −0.55 (*p* < 0.05).

**Table 4:** Subgroup analyses of symptom duration, anxiety, and depression.

**Variable**	**Mean DHI score (SD)**	**Mean EQ-5D score (SD)**	**Regression coefficient (95% CI)**	***p-*value**
Symptom duration (< 1 year)	30.42 (7.54)	0.81 (0.12)	−0.35 (−0.50, −0.20)	0.001
Symptom duration (>1 year)	45.62 (9.31)	0.61 (0.10)	−0.45 (−0.60, −0.30)	0.001
HADS anxiety (low: 0–7)	28.45 (6.75)	0.85 (0.11)	−0.20 (−0.35, −0.10)	0.045
HADS anxiety (moderate: 8–10)	38.52 (8.13)	0.72 (0.14)	−0.32 (−0.45, −0.20)	0.032
HADS anxiety (high: 11–21)	48.23 (10.34)	0.62 (0.15)	−0.52 (−0.65, −0.40)	0.001
HADS depression (Low: 0–7)	29.34 (6.85)	0.84 (0.10)	−0.25 (−0.40, −0.15)	0.039
HADS depression (moderate: 8–10)	40.12 (7.89)	0.71 (0.13)	−0.38 (−0.50, −0.25)	0.028
HADS depression (High: 11–21)	49.78 (9.45)	0.60 (0.14)	−0.55 (−0.70, −0.40)	0.001

DHI, Dizziness handicap inventory; EQ-5D, EuroQol-5 dimension; HADS, Hospital Anxiety and depression scale; SD, Standard deviation; CI, Confidence Interval.

**Figure 3:**
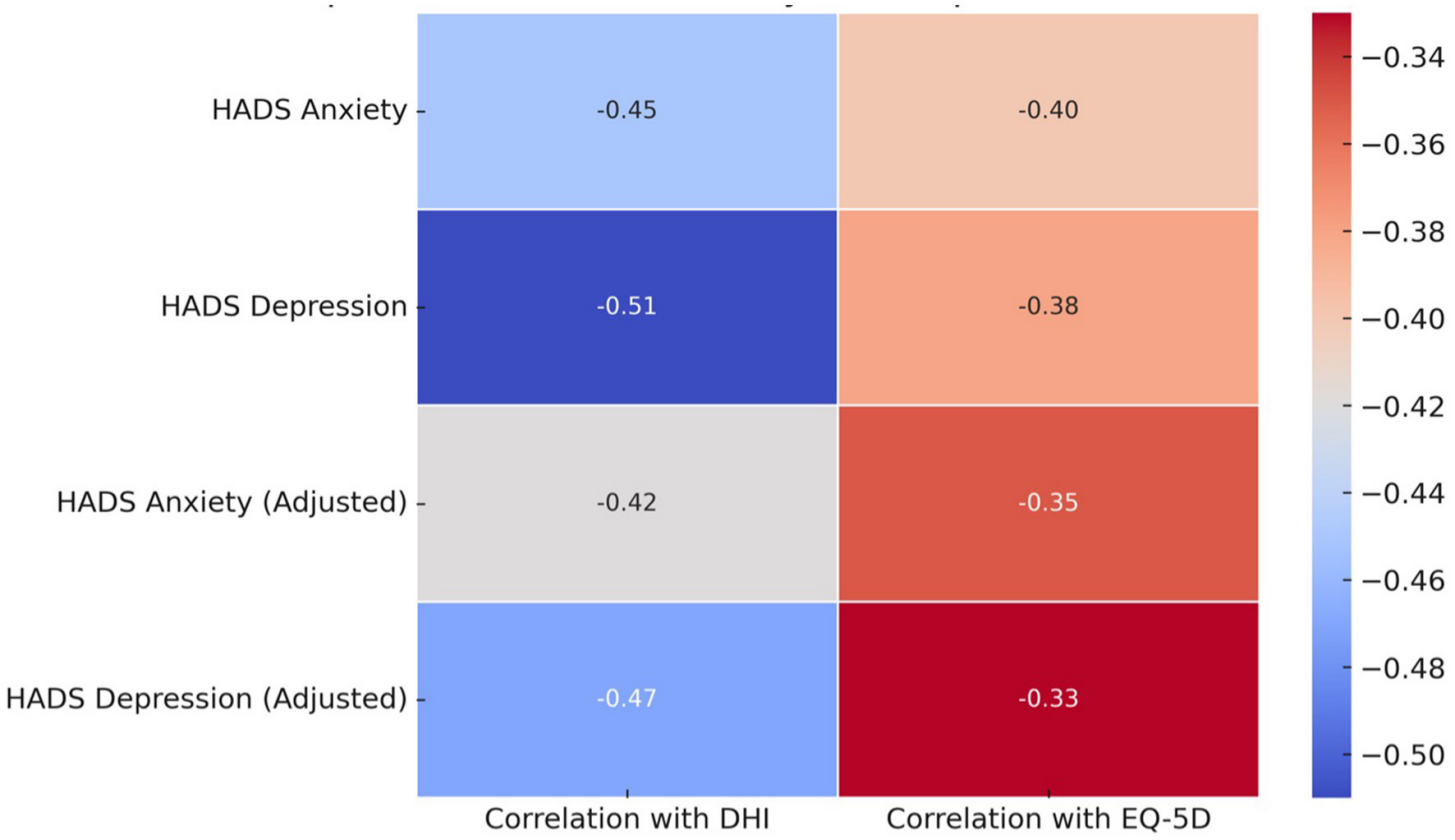
Heatmap of correlations between anxiety, depression, DHI, and EQ-5D in VRT patients.

The correlation and regression analysis revealed that both anxiety and depression were significantly associated with dizziness severity and functional outcomes in patients undergoing VRT (Table 5; Figure 3). HADS Anxiety and HADS Depression scores showed moderate negative correlations with DHI scores (*r* = −0.45 and *r* = −0.51, respectively), indicating that higher anxiety and depression were associated with worse dizziness-related handicap. Similarly, these variables were negatively correlated with EQ-5D scores, reflecting poorer QoL. In the adjusted regression models, both anxiety and depression remained significant independent predictors of DHI and EQ-5D outcomes, with depression showing a slightly stronger impact (*p* < 0.001). These findings suggest that addressing mental health factors is crucial in improving the effectiveness of VRT for PPPD patients. At 12 weeks post-therapy, the improvements in EQ-5D and DHI scores observed immediately post-VRT were largely sustained, with mean EQ-5D scores remaining at 0.71 and mean DHI scores showing a slight decline to 39.12 (*p* = 0.052). The adjusted regression model showed that depression had a slightly stronger effect on DHI outcomes (*B* = −0.44, adjusted *R*^2^ = 0.35, *p* < 0.001) compared to anxiety (*B* = −0.38, adjusted *R*^2^ = 0.28, *p* = 0.002).

**Table 5:** Correlation and regression analysis of anxiety and depression with dizziness and functional outcomes in VRT patients.

**Variable**	**Pearson correlation with DHI**	**Pearson correlation with EQ-5D**	**Regression coefficient (B)**	**95% CI (lower)**	**95% CI (upper)**	***p-*value**
HADS anxiety	−0.45	−0.40	−0.38	−0.55	−0.28	0.002
HADS depression	−0.51	−0.38	−0.44	−0.63	−0.37	< 0.001
HADS anxiety (Adjusted)	−0.42	−0.35	−0.33	−0.51	−0.21	0.004
HADS depression (Adjusted)	−0.47	−0.33	−0.41	−0.59	−0.32	< 0.001

HADS, Hospital anxiety and depression scale; DHI, Dizziness handicap inventory; EQ-5D, EuroQol-5 dimension; B, Regression coefficient.

## 4 Discussion

The objective of the study was to assess the effect of VRT on QoL and dizziness-related handicap, as well as to investigate the significance of anxiety and depression in patients with a diagnosis of PPPD. The results showed that QoL was substantially better for patients who underwent VRT than for those who did not, suggesting that the intervention helped improve quality of life by allowing them to perform day-to-day activities better. The association of longer symptom duration with more severe dizziness-related handicaps highlights the need for early intervention. Moreover, it was found that anxiety and depression were independent predictors of dizziness severity and QoL; higher anxiety and depression levels were associated with worse outcomes. This finding highlights the nuanced effects of VRT and the role of mental health factors in maximizing treatment efficacy in PPPD patients.

The observed significant improvement in QoL in patients who underwent VRT compared to those who did not can be attributed to the direct effects of VRT on mitigating the symptoms of PPPD (23). VRT primarily aims to enhance vestibular compensation, reducing dizziness-related discomfort, which in turn allows patients to resume daily activities with fewer limitations (24). The improvement in EQ-5D scores in the VRT group, alongside the significant reduction in DHI scores, suggests that patients were able to regain functional abilities and experience less dizziness and related anxiety (25). The observed DHI scores in our study were lower than the threshold (>60) commonly reported for functional disorders in the literature. This discrepancy may reflect the heterogeneity of our study population, which included participants with varying severities of PPPD, including early-stage and less severe cases. Furthermore, differences in referral patterns, healthcare settings, and population demographics could contribute to these variations. These findings underscore the need for individualized assessment and intervention strategies tailored to the diverse presentations of PPPD. The correlation between shorter symptom duration and better DHI scores also supports the idea that early intervention through VRT may prevent the chronicity of symptoms and their long-term impact on QoL (25). Moreover, the significant influence of mental health factors like anxiety and depression, as shown in the regression analysis, indicates that addressing psychological comorbidities in PPPD patients is essential to optimizing the outcomes of VRT (26).

Previous studies align with these findings. Trinidade et al. (5) demonstrated that VRT significantly improves all aspects of daily life, including emotional and functional components, in PPPD patients (5). Their results, which showed a reduction in DHI scores post-VRT, are consistent with the present study's findings, confirming VRT as a valuable treatment for managing dizziness and its related handicaps (27). Similarly, research by Molnár et al. (28) indicated that anxiety and depression significantly affect dizziness severity and QoL in patients with vestibular disorders, further validating the importance of psychological interventions alongside physical therapy (28). Collectively, these findings reinforce the role of VRT in both reducing the physical symptoms of PPPD and enhancing the overall well-being of patients by addressing the psychological burden associated with chronic dizziness (29).

While statistically significant reductions in DHI scores were observed post-VRT, the clinical relevance of these changes is underscored by comparing them to established thresholds such as the Minimal Detectable Change (MDC) and the Minimal Clinically Important Difference (MCID). Existing literature suggests an MCID for the DHI of ~10–18 points (30, 31). In our study, the mean reduction in DHI scores exceeded this range, demonstrating both statistical and clinically meaningful improvements in dizziness-related handicap, which are relevant to patient outcomes and clinical practice (31).

The results indicate that patients with PPPD who have had symptoms for over 1 year experience significantly higher dizziness-related handicaps compared to those with a shorter duration of symptoms (32). This can be attributed to the progressive nature of PPPD, where prolonged exposure to dizziness and balance disturbances leads to the accumulation of functional limitations and psychological burdens. Chronic dizziness exacerbates anxiety, reduces patients' confidence in performing daily activities, and may lead to a cycle of physical deconditioning, further contributing to worse DHI scores (33). Moreover, patients with longer symptom duration are likely to have developed maladaptive behaviors, such as avoiding environments that trigger dizziness, leading to greater impairments in social and occupational functioning (34). These findings underscore the need for early diagnosis and intervention to prevent the long-term disability associated with chronic dizziness (34).

Previous studies have reported similar trends, further validating the observed results (23). Schmid et al. (29) found that PPPD patients with a longer duration of symptoms exhibit greater dizziness handicaps and a reduced QoL, which parallels the significant differences observed in DHI scores in this study (29). Similarly, Teh et al. (23) emphasized that chronic dizziness is associated with higher levels of anxiety and functional limitations, which aligns with the present findings (23). These studies suggest that the severity of dizziness-related handicaps is not only a function of symptom intensity but also of symptom duration, further supporting the importance of timely intervention and the management of psychological comorbidities in patients with chronic vestibular disorders (29).

The significant associations between anxiety, depression, and dizziness severity, as well as their impact on functional outcomes, can be explained by the interconnected nature of psychological and vestibular disorders (35). Anxiety and depression are known to exacerbate the perception of dizziness and its associated handicaps by heightening patients' sensitivity to sensory stimuli, contributing to a vicious cycle of symptom amplification (36). Patients with higher anxiety may develop an increased fear of dizziness episodes, which leads to avoidance behaviors and further functional limitations, as evidenced by the strong negative correlations with DHI scores in this study. Similarly, depression reduces patients' motivation and ability to engage in physical rehabilitation, further impeding their progress (37). The regression analysis also indicates that depression had a slightly stronger influence on both DHI and EQ-5D scores, highlighting its critical role in determining overall functional outcomes in patients undergoing vestibular rehabilitation.

These findings are consistent with previous research that emphasizes the influence of psychological factors on vestibular disorders (38, 39). For instance, studies by Whitney et al. (38) and Staab et al. (39) demonstrated that anxiety and depression are common comorbidities in patients with vestibular dysfunction, often leading to worse functional outcomes (38). Similarly, Carren Sui-Lin Teh et al. (40) found that the severity of depression was closely related to greater dizziness handicap and poorer QoL in PPPD patients, supporting the present study's findings that depression has a significant independent effect on both DHI and EQ-5D outcomes (40). These results collectively suggest that comprehensive treatment approaches addressing both the physical and psychological aspects of dizziness are crucial for optimizing the effectiveness of VRT and improving patient outcomes (41).

We acknowledge that the relationship between anxiety, depression, and dizziness severity in PPPD may not be unidirectional. In addition to anxiety and depression potentially exacerbating dizziness severity through heightened symptom perception, it is equally plausible that patients with more severe PPPD symptoms are at increased risk of developing psychological distress (42). Greater dizziness severity may lead to substantial functional impairments, triggering or worsening anxiety and depression due to limitations in daily activities and social participation (43). This bidirectional relationship aligns with previous findings in the literature and highlights the complex interplay of psychological and physical factors in PPPD (44). Future studies employing longitudinal designs are essential to disentangle these relationships and guide holistic treatment strategies.

### 4.1 Clinical significance

The clinical significance of this study lies in its demonstration that VRT is an effective intervention for improving QoL and reducing dizziness-related handicaps in patients with PPPD. Importantly, the findings highlight that early intervention is key, as patients with a shorter duration of symptoms experienced less severe dizziness handicaps (45). Moreover, the study underscores the critical role of addressing psychological factors, particularly anxiety and depression, in enhancing VRT outcomes. The significant negative impact of these mental health comorbidities on both dizziness severity and QoL suggests that a holistic, multidisciplinary approach is necessary for managing PPPD (5). By incorporating psychological support alongside VRT, clinicians can improve patient adherence, reduce symptom chronicity, and optimize functional recovery, ultimately enhancing the overall effectiveness of treatment in this population (46).

### 4.2 Limitations of the study

Despite its contributions, this study has several limitations. First, the use of a non-VRT observational cohort in the retrospective comparisons invited bias since this cohort was not prospectively matched to the VRT cohort for comparison. Although baseline characteristics were similar across arms, the absence of randomization precludes causal conclusions. Also, the use of subjective measures, like DHI, may affect results. Prospective randomized designs and objective assessment tools in larger studies should be used to confirm and extend these findings. This study has shown that patients with PPPD experience numerous mental health-related benefits following the commencement of rehabilitation through VRT, suggesting that the intervention acts upon psychological chronic components that require overarching management to reach therapeutic benefit. The importance of synthesizing psychological support and facilitating early intervention strategies is critical given the complexity of this relatively rarely diagnosed condition. Also, this study utilized a pre-post design within the VRT group, complemented by baseline comparisons with a retrospectively identified non-VRT observational group. While this approach offers valuable real-world insights, the absence of prospective matching or random assignment limits the generalizability of the findings. The retrospective non-VRT group provided context for symptom severity in untreated patients but was not a prospective control, and this limitation should be considered when interpreting the results.

## 5 Conclusions

In conclusion, this study demonstrates that VRT significantly improves QoL and reduces dizziness-related handicaps in patients with PPPD. Early intervention with VRT is particularly beneficial, as patients with a shorter symptom duration experience less severe functional impairment. Moreover, anxiety and depression were found to be independent predictors of treatment outcomes, underscoring the importance of addressing mental health comorbidities to maximize the effectiveness of VRT. These findings suggest that a comprehensive treatment approach, incorporating both physical and psychological interventions, is essential for optimizing clinical outcomes in PPPD patients.

## Data Availability

The original contributions presented in the study are included in the article/supplementary material, further inquiries can be directed to the corresponding author.
